# Sequential MRI Evaluation of Lymphatic Abnormalities over the Course of Fontan Completion

**DOI:** 10.1148/ryct.230315

**Published:** 2024-05-30

**Authors:** Benjamin Kelly, Sheyanth Mohanakumar, Brooke Ford, Christopher L. Smith, Erin Pinto, David M. Biko, Vibeke E. Hjortdal, Yoav Dori

**Affiliations:** From the Departments of Cardiothoracic Surgery (B.K.) and Radiology (S.M.), Aarhus University Hospital, Palle Juul-Jensens Boulevard 99, 8200 Aarhus N, Denmark; Division of Cardiology (B.K., B.F., C.L.S., E.P., Y.D.) and Department of Radiology (D.M.B.), Children's Hospital of Philadelphia, Philadelphia, Pa; and Department of Cardiothoracic Surgery, Copenhagen University Hospital–Rigshospitalet, Copenhagen, Denmark (V.E.H.).

**Keywords:** Congenital Heart Disease, Glenn, Fontan, Lymphatic Imaging, Cardiovascular MRI

## Abstract

**Purpose:**

To evaluate lymphatic abnormalities before and after Fontan completion using noncontrast lymphatic imaging and relate findings with postoperative outcomes.

**Materials and Methods:**

This study is a retrospective review of noncontrast T2-weighted lymphatic imaging performed at The Children's Hospital of Philadelphia from June 2012 to February 2023 in patients with single ventricle physiology. All individuals with imaging at both pre-Fontan and Fontan stages were eligible. Lymphatic abnormalities were classified into four types based on severity and location of lymphatic vessels. Classifications were compared between images and related to clinical outcomes such as postoperative drainage and hospitalization, lymphatic complications, heart transplant, and death.

**Results:**

Forty-three patients (median age, 10 years [IQR, 8–11]; 20 [47%] boys, 23 [53%] girls) were included in the study. Lymphatic abnormalities progressed in 19 individuals after Fontan completion (distribution of lymphatic classifications: type 1, 23; type 2, 11; type 3, 6; type 4, 3 vs type 1, 10; type 2, 18; type 3, 10; type 4, 5; *P* = .04). Compared with individuals showing no progression of lymphatic abnormalities, those progressing to a high-grade lymphatic classification had longer postoperative drainage (median time, 9 days [IQR, 6–14] vs 17 days [IQR, 10–23]; *P* = .04) and hospitalization (median time, 13 days [IQR, 9–25] vs 26 days [IQR, 18–30]; *P* = .03) after Fontan completion and were more likely to develop chylothorax (12% [three of 24] vs 75% [six of eight]; *P* < .01) and/or protein-losing enteropathy (0% [0 of 24] vs 38% [three of eight]; *P* < .01) during a median follow-up of 8 years (IQR, 5–9). Progression to any type was not associated with an increased risk of adverse events.

**Conclusion:**

The study demonstrated that lymphatic structural abnormalities may progress in select individuals with single ventricle physiology after Fontan completion, and progression of abnormalities to a high-grade classification was associated with worse postoperative outcomes.

**Keywords:** Congenital Heart Disease, Glenn, Fontan, Lymphatic Imaging, Cardiovascular MRI

*Supplemental material is available for this article.*

Published under a CC BY 4.0 license.

## Graphical abstract

**Figure:**
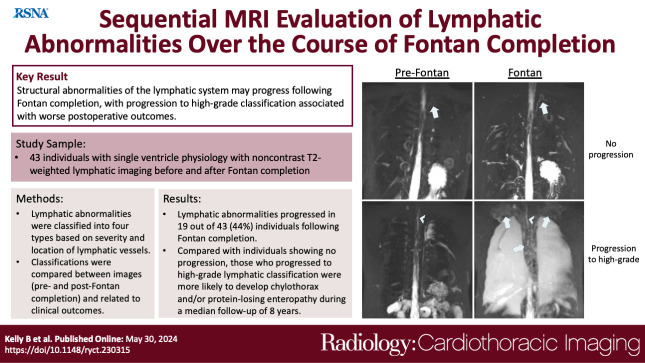

**Summary**

The lymphatic system in the single ventricle population is dynamic, and abnormalities progress after Fontan completion. Progression to a high-grade lymphatic classification is associated with poor postoperative outcomes.

**Key Points**

■ With the use of an established classification scheme, signal abnormalities at lymphatic imaging were shown to be dynamic and progress over the course of Fontan completion.
■ Progression to a high-grade classification was associated with a poor postoperative outcome during a median 8 years (IQR, 5–9) of follow-up when compared with individuals displaying no progression.
■ For risk monitoring, the clinician may want to consider lymphatic imaging more routinely as part of Fontan assessment or reassessment.

## Introduction

The single ventricle population remains one of the most challenging congenital heart defect populations. Although perioperative mortality and 30-year survival have both improved dramatically, reaching 1% and 85%, respectively, morbidity throughout life remains substantial (1–3). An estimated 40% of the single ventricle population will experience Fontan failure over the time span of 2 decades (4,5). Early identification of individuals at risk for deterioration and Fontan failure is an important challenge in this growing population (3).

The lymphatic system is intertwined with the cardiovascular circulation. In the single ventricle population, failure of the lymphatic system to maintain fluid homeostasis and prevent inappropriate leakage is termed *lymphatic insufficiency*. It may manifest itself as either protein-losing enteropathy (PLE), plastic bronchitis (PB), or persistent pleural effusions (6). New imaging modalities and promising options of treatment have improved knowledge of this unknown system (7–12). Lymphatic insufficiency is now thought to be the culmination of multiple hits by a number of disposing factors. Venous congestion is central and inevitable in single ventricle physiology. It increases production of lymph fluid while simultaneously worsening the lymphovenous gradient and challenging lymphatic return. Lymphatic congestion and increased lymphatic afterload may dilate lymphatic vessels, cause incompetent valves, and reduce lymphatic pumping function (13–15). Finally, an abnormal lymphatic architecture is associated with poor outcomes when observed in utero and at every one of the subsequent stages leading to Fontan completion (16–22). Accordingly, lymphatic imaging has been introduced as an integral part of cardiac MRI evaluations of individuals with single ventricle physiology.

It is unknown if structural lymphatic changes progress over the course of Fontan completion. In terms of risk monitoring, the value of repeated lymphatic imaging remains speculative. The current study aimed to evaluate lymphatic abnormalities at lymphatic MRI before and after Fontan completion and relate findings with outcome parameters of lymphatic insufficiency, transplant, and death.

## Materials and Methods

This Health Insurance Portability and Accountability Act–compliant study was approved by The Children's Hospital of Philadelphia Institutional Review Board, and the need for informed consent was waived (IRB 21–019584).

### Study Design and Sample

This was a retrospective review of serial lymphatic imaging of patients with single ventricle physiology at The Children's Hospital of Philadelphia performed from June 2012 to February 2023. To be included in the study, patients were required to have available noncontrast, three-dimensional, T2-weighted sampling perfection with application-optimized contrasts using different flip-angle evolution (SPACE) sequences performed before and after Fontan completion (ie, when staged with a superior cavopulmonary connection and a total cavopulmonary connection). In the case of multiple T2-weighted SPACE sequences at either stage, the imaging giving the longest follow-up period before any potential lymphatic intervention or heart transplant was chosen. An unknown number of the included patients have been described by Biko et al (19) in a study on the relationship between pre-Fontan imaging and postoperative outcome.

Noncontrast lymphatic imaging has been a part of the standard protocol for patients with single ventricle physiology undergoing cardiac MRI at The Children's Hospital of Philadelphia since 2012. Although cardiac MRI is routinely performed before Fontan completion, the indication for cardiac MRI after Fontan completion is evaluated on a case-by-case basis by the treating physicians.

### Cardiac and Lymphatic MRI Protocol

The T2-weighted SPACE sequence for noncontrast lymphatic imaging has been previously described (18,19). The sequence is available on all commercial Siemens machines and from other vendors under different names. In short, images were obtained in the coronal orientation with a 1.5-T MRI magnet (MAGNETOM Avanto; Siemens). The following parameters were used: matrix, 256 × 256; field of view, 300–450; repetition time, 2500 msec; echo time, 650 msec; flip angle, 140°; and voxel size, 1.0 × 1.0 × 1.0 to 1.3 × 1.3 × 1.3 mm. All images included the neck and chest region, with the most caudal cutoff of the abdominal region dependent on patient size. The acquisition time was approximately 4–6 minutes, depending on patient size. For cardiac MRI, ventricular volumetry was performed with balanced steady-state free precession short-axis cine images, and blood flow was assessed with through-plane velocity-encoded phase-contrast images in vessels of interest. The area of the Fontan pathway was calculated based on the sagittal (*a*) and coronal (*b*) pathway diameters (area = π ∙ (*a* + *b*)/2). Atrioventricular valve regurgitation fraction was estimated by evaluating both the atrioventricular-valve inflow versus aortic outflow and the cine stroke volume versus aortic outflow (23). A regurgitant fraction of less than 25% was considered mild, 25%–45% as moderate, and more than 45% as severe.

### Lymphatic MRI Analysis

**Figure 1:**
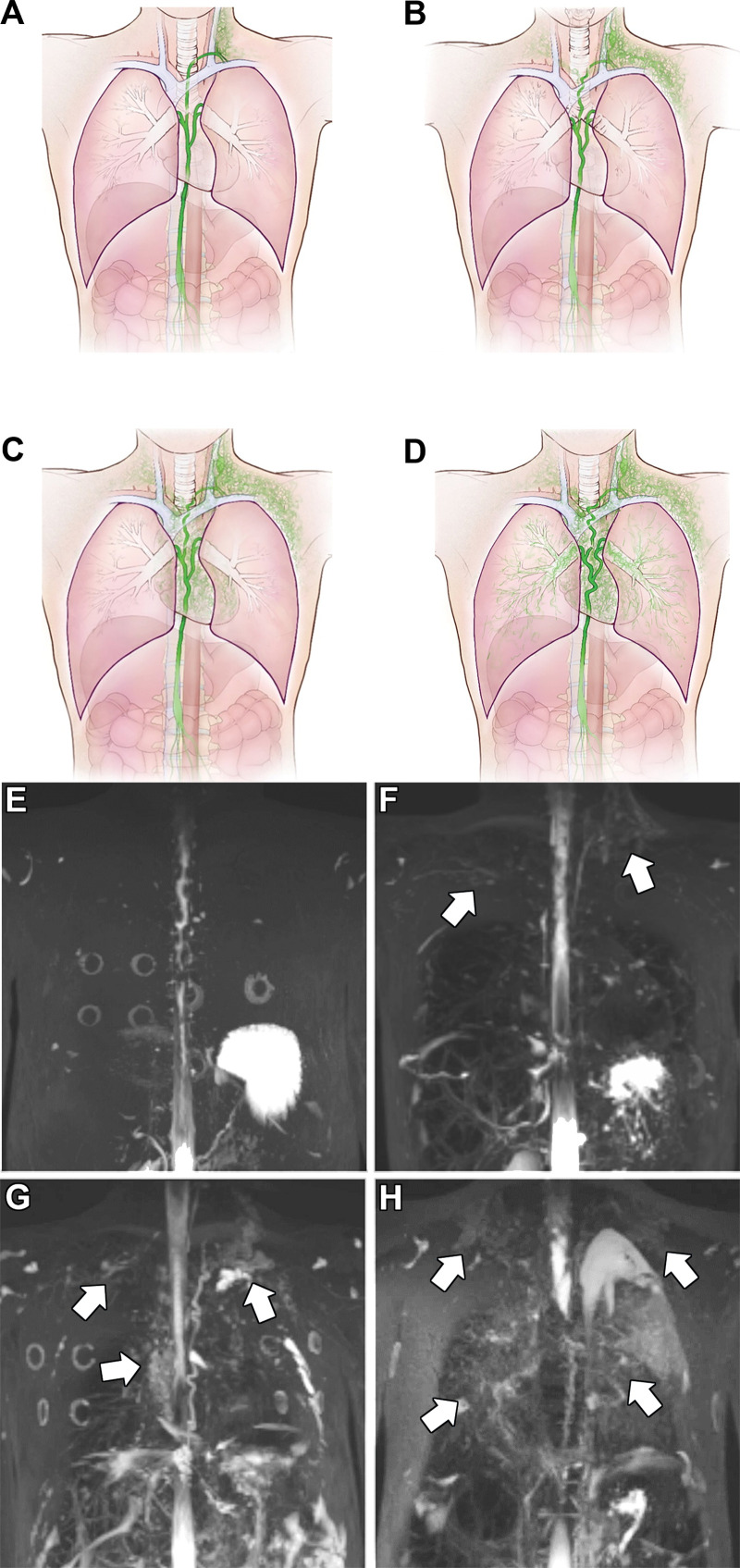
**(A–D)** Schematic representation of lymphatic classification types 1–4. **(A)** Type 1. Unilateral supraclavicular minimally increased signal intensity. **(B)** Type 2. Increased signal intensity within the bilateral supraclavicular region without extension into the mediastinum. **(C)** Type 3. Increased signal intensity in the supraclavicular regions and extending into the mediastinum. **(D)** Type 4. Increased abnormal signal intensity in the bilateral supraclavicular regions extending into the mediastinum and with an interstitial pattern into the lungs. (Reprinted, with permission, from reference 19.) **(E–H)** Representative noncontrast T2-weighted lymphatic imaging of types 1–4 of individuals with a Fontan circulation. **(E)** T2-weighted lymphatic image in a 6-year-old boy with Fontan circulation, with changes in signal abnormalities corresponding to type 1. The patient did not develop complications in the follow-up period. **(F)** T2-weighted lymphatic image in a 7-year-old girl with Fontan circulation, with signal abnormalities in bilateral supraclavicular regions (arrows) corresponding to type 2. The patient did not develop complications in the follow-up period. **(G)** T2-weighted lymphatic image in a 9-year-old boy with Fontan circulation, with signal abnormalities in bilateral supraclavicular regions and in the mediastinum (arrows) corresponding to type 3. The patient developed ascites during the follow-up period. **(H)** T2-weighted lymphatic image in a 4-year-old boy with Fontan circulation, signal abnormalities presenting bilaterally in the supraclavicular regions, in the mediastinum and in the lungs (arrows) corresponding to type 4. The patient was readmitted during the follow-up period for pleural effusions and chylothorax, plastic bronchitis, ascites, lymphatic intervention, and ultimately heart transplant.

Two analysts (B.K. and S.M., each with 5 years of experience evaluating lymphatic imaging) independently evaluated all lymphatic images while blinded to clinical outcomes. Images were classified in accordance with a previously published lymphatic classification system (18,19,22). For low-grade lymphatic changes, abnormal signal intensities were to be present in one (type 1) or both (type 2) supraclavicular regions. High-grade lymphatic changes were defined as supraclavicular abnormalities in combination with mediastinal signal intensity changes (type 3) or extension into both the mediastinum and with an interstitial pattern out into the lung parenchyma (type 4) (Fig 1). Analysis was conducted on multisection images acquired as described above and in the coronal plane only (Fig S1 describes the analysis). Interrater reliability was calculated based on the two initial evaluations. After independent evaluation, any discrepancies were discussed in plenum with a third evaluator (Y.D., a pediatric interventional cardiologist with 11 years of experience evaluating lymphatic imaging) until a final consensus was reached.

### Demographic, Clinical, and Hemodynamic Data

Medical records were retrospectively evaluated for demographic and clinical characteristics of included patients. Data from cardiac catheterization and cardiac MRI were selected and included with the same criteria as for the lymphatic MRI. If multiple catheterizations had been performed, the one nearest lymphatic imaging was chosen. The following outcomes were collected from the postoperative period: days of drainage, prolonged drainage defined as more than 14 days of drainage after Fontan completion, days of hospitalization, and readmission for any effusion. Diagnosis of chylothorax (triglyceride level > 110 mg/dL [1.24 mmol/L] or > 80% lymphocyte count in pleural fluid), PLE (spot α_1_-antitrypsin value > 54 mg/dL [9.9 μmol/L], or total clearance > 27 mL/24 h) or PB (confirmed by presence of casts or visualized lymphatic airway leakage) were all noted. Any lymphatic intervention (thoracic duct embolization, selective lymphatic duct embolization, and innominate vein turndown), Fontan takedown, heart transplant, and death were also recorded. For analysis, prolonged drainage; readmission for pleural effusions; diagnosis of chylothorax, PLE, or PB; any lymphatic intervention; transplant; or death were combined in a composite end point called *lymphatic events*. All covariates and demographic and outcome variables were reviewed until the time of final inclusion (March 1, 2023).

### Statistical Analysis

Included continuous variables are presented as means ± SDs for normally distributed data and medians (IQRs) for nonnormally distributed variables. Categorical variables are presented as numbers with percentages in parentheses. Individuals were grouped into low-grade lymphatic changes (types 1–2) or high-grade lymphatic changes (types 3–4) or were based on progression in grading between lymphatic images (status quo or worsened). Depending on the distribution, the unpaired *t* test or Wilcoxon signed rank test was used when comparing the characteristics and outcomes of patients in these groups. For categorical variables, the Fisher exact test was used to test for differences between groups. The Spearman rank correlation coefficient was used to test for correlations between variables. Interrater agreement for lymphatic classification was calculated using Cohen κ statistic coefficient. A κ statistic coefficient of 0.61 or more indicated substantial agreement and 0.75 or more excellent agreement. For all tests, *P* < .05 was considered statistically significant. All calculations were made using Stata, version 15.1 (Stata), or Prism, version 6 (GraphPad).

## Results

### Patient Characteristics

From the 3627 noncontrast lymphatic MRI studies in The Children's Hospital of Philadelphia imaging registries, we identified 43 patients (median age, 10 years [IQR, 8–11]; 23 [53%] girls and 20 [47%] boys) with single ventricle physiology who had available T2-weighted lymphatic imaging at both pre-Fontan and Fontan stages (Fig S2). The median ages at superior cavopulmonary connection and total cavopulmonary connection completion were 4 (IQR, 4–5) and 36 (IQR, 31–43) months, respectively. The median ages at pre-Fontan cardiac catheterization and cardiac MRI were 16 (IQR, 7–32) and 33 (IQR, 27–39) months, respectively. The median ages at Fontan-stage cardiac catheterization and cardiac MRI were 47 (IQR, 38–91) and 79 (IQR, 50–116) months, respectively. The median length of follow-up after Fontan completion was 8 years (IQR, 5–9), totaling 303 years of follow-up (Table 1).

**Table 1:**
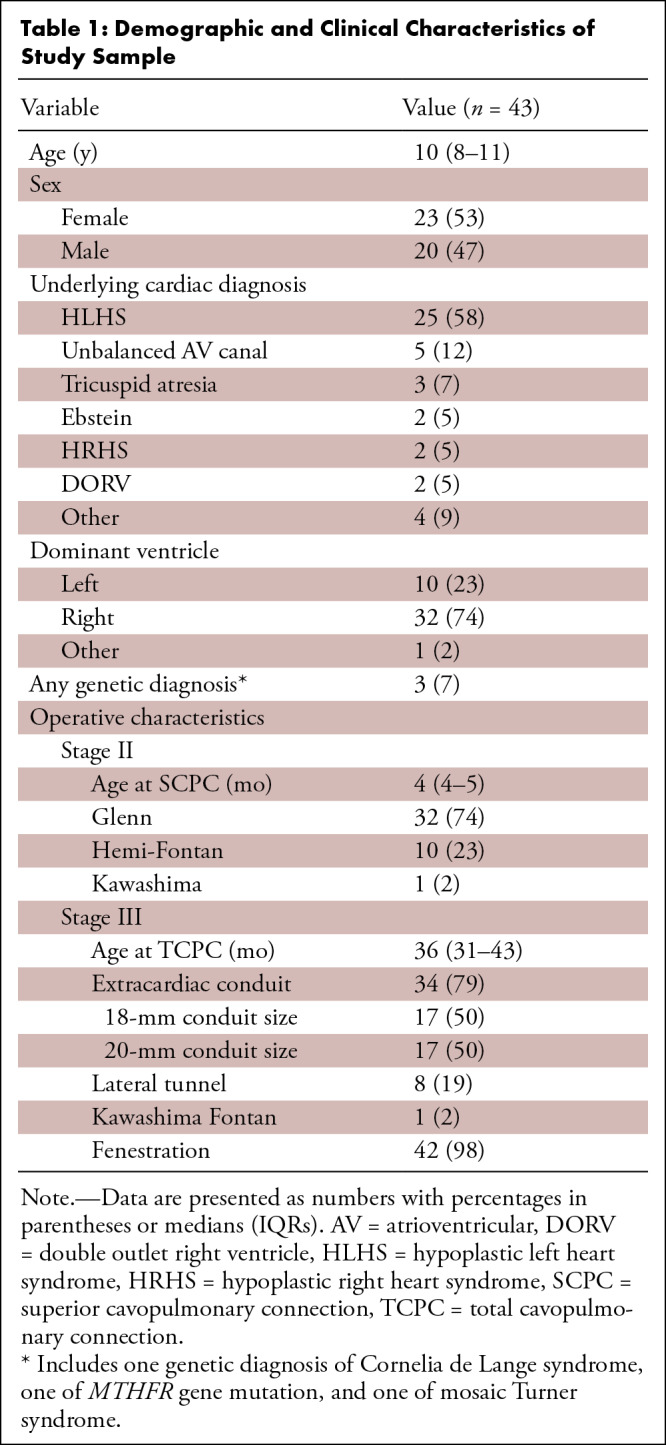
Demographic and Clinical Characteristics of Study Sample

### Interrater Agreement

The interrater agreement between the two initial evaluators was substantial, with a Cohen κ of 0.7. Discrepancies regarding types were between types 1 and 2 (*n* = 7), 2 and 3 (*n* = 4), 3 and 4 (*n* = 4), and 2 and 4 (*n* = 1). Discrepancies were reviewed with Y.D. until a final consensus was reached.

### Lymphatic Classification and Cardiac Catheterization and MRI

Of 43 patients, 36 (84%) underwent cardiac catheterization before Fontan completion. All patients underwent cardiac MRI before Fontan completion. Patients with high-grade lymphatic changes had greater pulmonary vascular resistance, greater pulmonary-to-systemic blood flow ratio, and less systemic venous return (*P* ≤ .03 for all comparisons).

After Fontan completion, 35 (81%) patients underwent cardiac catheterization. In individuals with high-grade lymphatic changes, the Fontan pressure, pulmonary artery pressure, and capillary wedge pressure were all significantly higher than in individuals with low-grade changes. At Fontan stage, 40 (93%) patients had cardiac indexes evaluated together with lymphatic imaging, and three (7%) had lymphatic imaging without additional measurements of cardiac function. Patients with high-grade lymphatic changes at Fontan imaging had a greater systemic-to-pulmonary collateral flow (*P* = .01) (Tables 2 and S1).

**Table 2:**
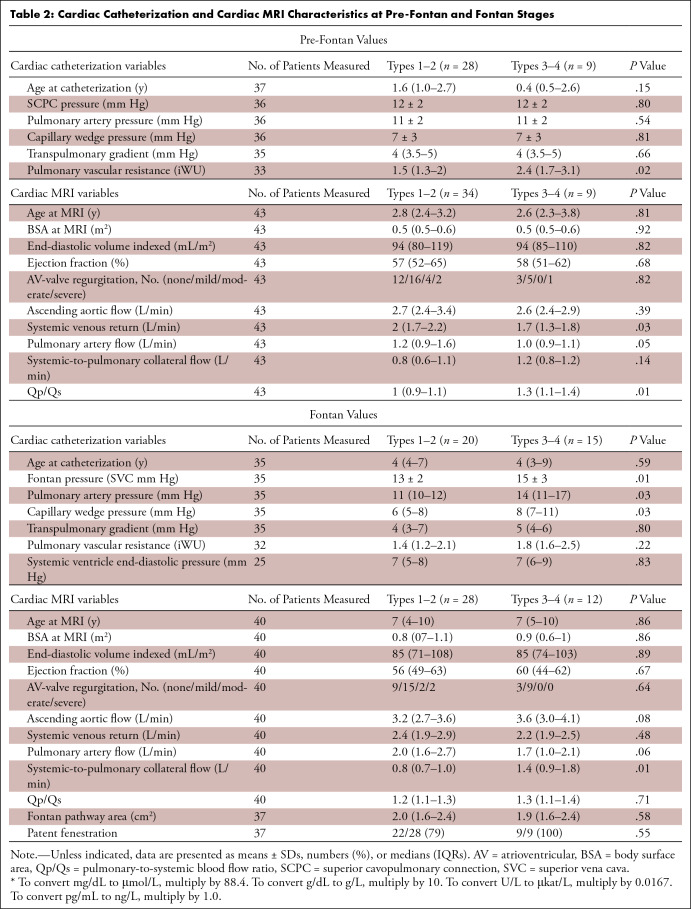
Cardiac Catheterization and Cardiac MRI Characteristics at Pre-Fontan and Fontan Stages

### Lymphatic Classification and Clinical Outcomes

Patients with high-grade lymphatic changes had longer postoperative drainage compared with patients with low-grade changes and longer postoperative hospitalizations after Fontan completion. When looking at associations between lymphatic classification and clinical outcomes in the years after Fontan completion, patients with high-grade lymphatic changes at pre-Fontan imaging were more likely to develop chylothorax or PB and were more likely to undergo lymphatic intervention or orthotopic heart transplantation. At Fontan stage, individuals with high-grade lymphatic abnormalities demonstrated a greater likelihood of prolonged effusions (required drainage > 2 weeks), chylothorax, PB, and PLE and a higher incidence of lymphatic intervention. Of patients with a high-grade classification at Fontan imaging, 12 of 15 (80%) experienced a lymphatic event postoperatively. This result was the case for only five of 28 (18%) patients with low-grade classification (*P* < .001). Excluding transplant and all-cause mortality from the composite end point did not change the results, as all patients with transplant or disease also experienced one or more lymphatic events. Lymphatic grading correlated significantly with the total number of lymphatic events (Spearman ρ: 0.74; *P* < .001 for Fontan imaging and 0.54; *P* < .001 for pre-Fontan imaging). Table 3 presents the proportions of patients experiencing clinical outcomes by lymphatic grading at either pre-Fontan or Fontan stage.

**Table 3:**
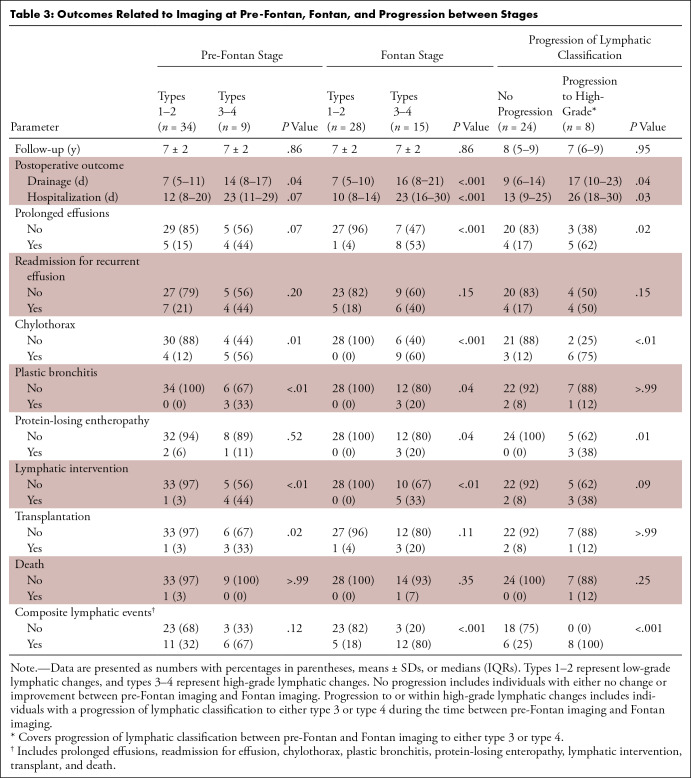
Outcomes Related to Imaging at Pre-Fontan, Fontan, and Progression between Stages

### Progression of Lymphatic Classification and Clinical Outcomes

**Figure 2:**
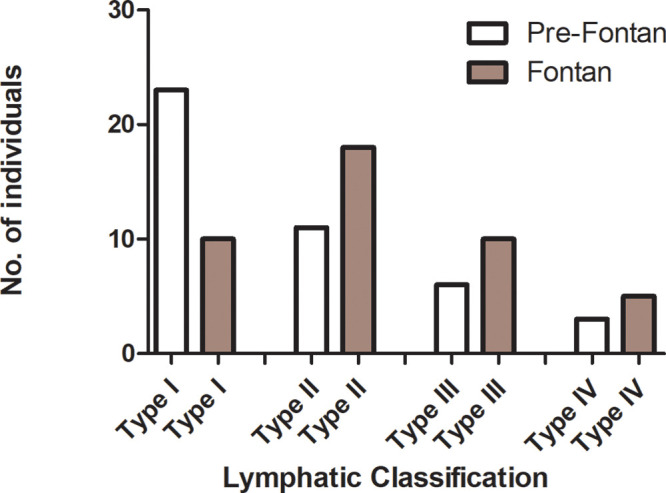
Bar chart illustrates the distribution of lymphatic classifications at pre-Fontan and Fontan stages. Distributions changed over time, with 19 (44%) of the 43 individuals progressing in lymphatic classification, two (5%) improving, and 22 (51%) displaying no change (*P* = .04).

**Figure 3:**
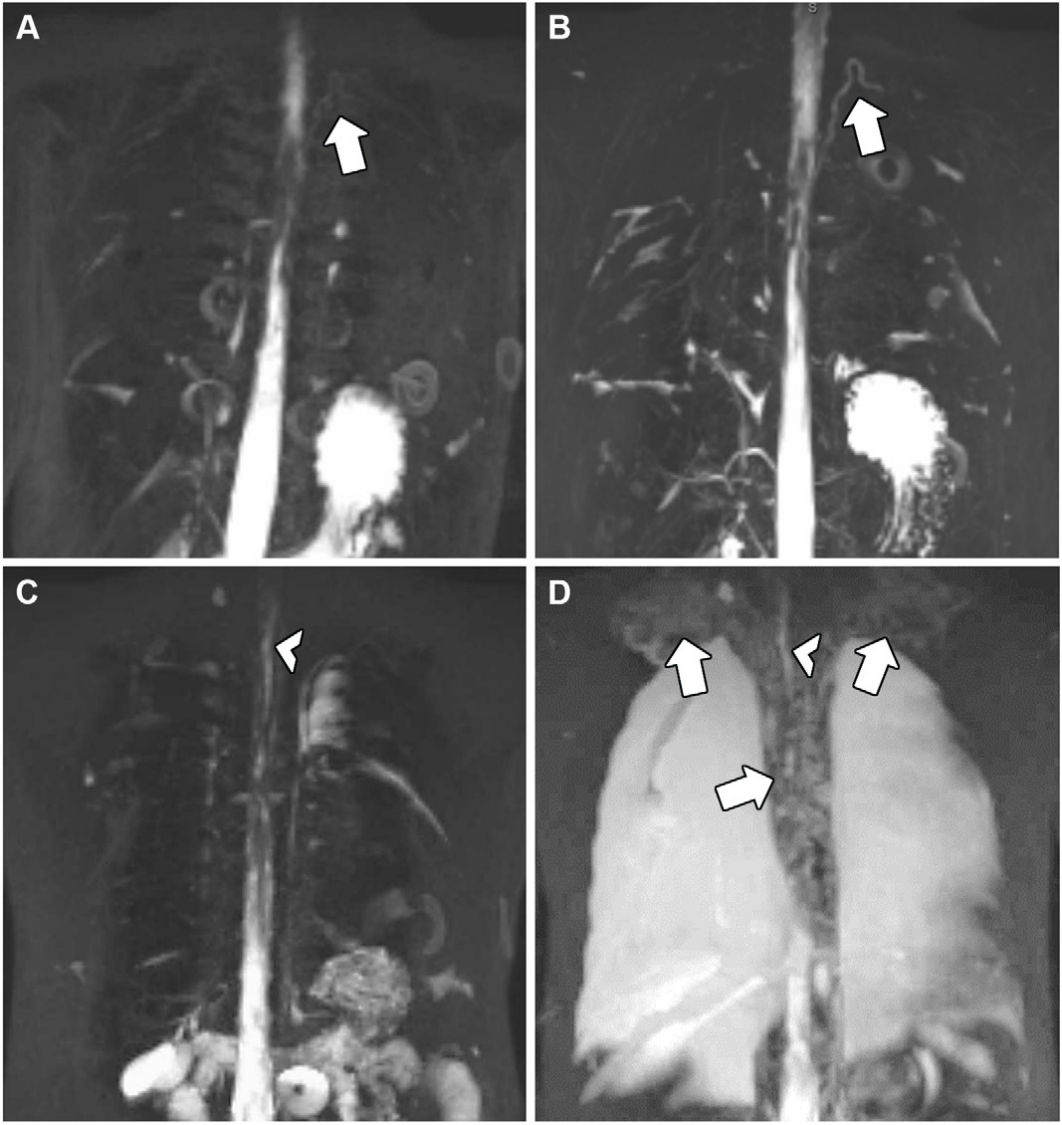
Maximal intensity projections of noncontrast lymphatic imaging in two individuals at pre-Fontan and Fontan stages. **(A)** Image in a 2-year-old girl at pre-Fontan stage and **(B)** image 2 years later at Fontan stage displaying no notable changes. The thoracic duct is visible and displaying a similar morphology on both images (arrows). The patient did not develop complications in the postoperative period. **(C)** Image at pre-Fontan staging in a 2-year-old girl displaying type 3 lymphatic classification (more mediastinal abnormal signal intensity visible on multisection image). **(D)** Image 2 months after Fontan completion in the same patient. The terminal part of the thoracic duct can be visualized on both images (arrowheads). A progression in abnormal signal intensity is especially apparent in the supraclavicular and mediastinal regions (arrows), imaging now categorized as type 4. The postoperative period was characterized by readmission for pleural effusion, chylothorax, and lymphatic intervention.

Lymphatic classification based on location and prominence of lymphatic vessels was dynamic and changed over the period between lymphatic images (*P* = .04) (Figs 2, 3). There was no greater prevalence of lymphatic events in the group experiencing progression of lymphatic classification compared with the group without progression (*P* > .06 for all comparisons). However, when comparing the group with stable imaging to the group progressing to a high-grade classification, the latter had more individuals who experienced a lymphatic event (*P* < .001) and a greater likelihood of each individual experiencing a greater number of events (*P* < .001). When looking independently at each outcome, progression to a high-grade classification was associated with longer drainage and hospitalization, chylothorax, and PLE (Table 3). In the current study sample, systemic right ventricle, extracardiac conduit size, Fontan pathway area (smallest quartile compared with remaining), and underlying congenital heart disease cause were not associated with a greater likelihood of progression or a greater classification at Fontan imaging (*P* > .22 for all comparisons).

## Discussion

In this study of sequential lymphatic imaging in 43 patients with single ventricle physiology, we reported the progression of lymphatic structural abnormalities in 44% of the examined patients over the course of Fontan completion. Although progression to a low-grade classification carried no increased risk, progression to a high-grade classification was associated with a longer period of postoperative drainage, longer postoperative hospitalization, chylothorax, and PLE in the follow-up period. For risk monitoring, the clinician may want to consider lymphatic imaging more routinely as part of Fontan assessment or reassessment, as changes may be dynamic and progress over the course of Fontan completion.

An increasing amount of attention is currently diverted into tailoring adequate and often extensive follow-up for the rapidly growing single-ventricle population. Tools that provide risk assessment and help with early recognition of challenges may help guide treatment. This study is unique in reporting structural changes in the lymphatic system over the course of Fontan completion. It adds to the growing amount of evidence supporting the efficacy of noncontrast lymphatic imaging for estimation of risk. In 18 of 43 (44%) of the patients included, the lymphatic classification progressed to a higher grade over the time between acquisitions of images. Previously, serial lymphatic imaging has been described in 33 individuals with single ventricle physiology after Fontan completion (24). Although many of the individuals experienced improvements (nine of 33 [27%]) or deterioration (six of 33 [18%]) of their lymphatic classification, the overall distribution did not change over the 4.5 years between image acquisitions. In another study using a threshold-based segmentation of high-intensity lymphatic areas on the maximum intensity projection of T2-weighted lymphatic MRI, Vaikom House et al (20) found no relationship between time since Fontan completion and lymphatic burden. Both studies speculated that progression of lymphatic abnormalities might take place after significant hemodynamic changes, such as those resulting from surgical procedures. With this study, we confirm that abnormalities do progress after the changes after Fontan completion. Progression to a high-grade lymphatic classification was associated with a longer period of postoperative drainage, longer postoperative hospitalization, chylothorax, and PLE in the follow-up period. The findings add important aspects to the understanding of the changes in the lymphatic system in the single ventricle population. In terms of risk monitoring, lymphatic imaging should be considered more routinely as part of Fontan assessment or reassessment, as findings may be dynamic and progress over the course of Fontan completion.

Previously, lymphangiectasia in utero or early changes after initial palliation have been associated with increased mortality (16,17,22). At pre-Fontan lymphatic imaging, high-grade changes are associated with longer drainage and longer postoperative stays after Fontan completion and a sixfold increase in risk for early complications (18,19). After Fontan completion, Vaikom House et al (20) also found a higher lymphatic burden in individuals developing PLE, recurrent effusions, or heart failure. Similarly, lymphatic area score was associated with secondary in-hospital treatment due to a number of lymphatic complications (21). In this study, high-grade lymphatic changes at pre-Fontan imaging were associated with increased length of drainage, chylothorax, plastic bronchitis, lymphatic intervention, and orthotopic heart transplantation postoperatively (*P* ≤ .04 for all comparisons). After Fontan completion, these findings became more pronounced. Increased duration of postoperative drainage, longer postoperative hospitalization, prolonged effusions, chylothorax, PB, PLE, and any lymphatic intervention during follow-up were associated with a high-grade lymphatic classification at Fontan imaging (*P* ≤ .04 for all comparisons). It should be noted that the inclusion criteria of sequential imaging excludes individuals unable to undergo Fontan completion, a group in which high-grade lymphatic changes on pre-Fontan images and lymphatic complications have previously been shown to be highly prevalent. Biko et al (19) reported failure to undergo Fontan completion to be rare among individuals with low-grade lymphatic changes; however, around 25% of all individuals with high-grade lymphatic changes on pre-Fontan images did not undergo Fontan completion.

No indexes of cardiac function or hemodynamics were associated with lymphatic classification at both pre-Fontan and Fontan stages. However, two findings deserve highlighting: the higher Fontan pressure and the greater systemic-to-pulmonary collateral burden found among patients with high-grade lymphatic classifications. Interestingly, both high central venous pressure and aortopulmonary collaterals have been associated with the development of a lymphatic insufficiency such as PLE (25,26). PLE rarely develops before the increase in inferior vena cava pressure caused by Fontan completion. It is interesting that all three patients who developed PLE during follow-up also displayed progression to high-grade lymphatic abnormalities (19,22,27). Irrespective of lymphatic grading, systemic-to-pulmonary collaterals are associated with increased duration of drainage and length of stay after Fontan completion (28,29). Although systemic-to-collateral flow correlated with lymphatic classification (*P* < .01), no correlation was found between systemic-to-collateral flow and postoperative drainage or hospitalization in our study (*P* > .13 for all comparisons). There is a known overlap in the signaling leading to both lymphatic vessel and blood vessel growth, maintenance, and proliferation (30,31). It is unknown if the mechanisms leading to the development of lymphatic collaterals share an overlap with the mechanisms leading to systemic-to-pulmonary collaterals.

Our study had several limitations. This is a retrospective follow-up study with inherent limitations, including being unable to determine causality between lymphatic changes and outcomes. All imaging and accompanying workup included in the current study were done at the same tertiary care center. Any follow-up for relevant outcomes at other centers may thus not be included in the study and is a source of potential bias. In addition, there is selection bias related to multiple aspects of the study. Cardiac MRI, including lymphatic imaging, is standard of care at our institution before Fontan completion. However, the second imaging acquisition at Fontan stage is performed only on clinical indication and when deemed necessary by the treating physicians (Fig S3). Accordingly, patients included may display a higher rate of complications, as those without complications are unlikely to be referred for imaging in the first years after Fontan completion. Additionally, the requirement of study individuals to undergo lymphatic imaging after Fontan completion excludes individuals failing to progress that far. Finally, no widely accepted method for the evaluation of lymphatic changes in the single ventricle population exists. Thus, studies apply individually developed methods to assess lymphatic architecture. Although findings point in a similar direction, the differences in method prevent direct comparison.

In conclusion, we report the structural abnormalities of the lymphatic system to be dynamic and progress in select individuals over the course of Fontan completion. Progression to a high-grade classification was associated with poor outcomes in the form of a longer period of postoperative drainage, longer postoperative hospitalization, and a greater risk of developing chylothorax and/or PLE in the follow-up period. For risk monitoring, the clinician may want to consider lymphatic imaging more routinely as part of Fontan assessment and reassessment. Future studies are needed to understand the driving force behind these lymphatic changes and their implications on single ventricle–associated pathophysiology.
